# Healthcare professionals’ perceptions of challenges in vaccine communication and training needs: a qualitative study

**DOI:** 10.1186/s12875-024-02509-y

**Published:** 2024-07-20

**Authors:** Dawn Holford, Emma C. Anderson, Aishmita Biswas, Amanda Garrison, Harriet Fisher, Emeline Brosset, Virginia C. Gould, Pierre Verger, Stephan Lewandowsky

**Affiliations:** 1 School of Psychological Science, University of Bristol, 12A Priory Road, Bristol, BS8 1TU United Kingdom; 2 Bristol Medical School, University of Bristol, Bristol, United Kingdom; 3 Faculté Des Sciences Médicales Et Paramédicales, Southeastern Health Regional Observatory (Observatoire Régional de La Santé, ORS) PACA, Marseille, France; 4 Bristol Medical School, National Institute for Health Research Health Protection Research Unit (NIHR HPRU) in Behavioural Science and Evaluation (BSE) University of Bristol, Bristol, United Kingdom; 5 Unité des Virus Émergents (UVE: Aix-Marseille Univ, Università di Corsica, IRD 190, Inserm 1207, IRBA), Marseille, France; 6 Department of Psychology, University of Potsdam, Potsdam, Germany; 7 School of Psychological Science, University of Western Australia, Perth, Australia

**Keywords:** Vaccine communication, Healthcare professionals, Skills training, Vaccine hesitancy, Vaccine confidence

## Abstract

**Background:**

Healthcare professionals (HCPs) can play an important role in encouraging patients and their caregivers to be vaccinated. The objective of this qualitative study was to investigate HCPs’ perspectives on challenges in vaccine communication and unmet training needs in this domain.

**Methods:**

Semi-structured interviews were conducted with 41 HCPs (mainly nurses and physicians) with vaccination roles (23 in England; 18 in France), gathering information on: (1) HCPs’ approach to vaccine conversations with patients; (2) Challenges of communicating about vaccines; (3) Vaccine-related training and learning resources available to HCPs, and; (4) HCPs’ training needs around vaccine communication.

**Results:**

HCPs described a range of communication experiences that indicated insufficient time, information, and skills to confidently navigate difficult conversations with vaccine-hesitant patients. Communication skills were especially important to avoid conflict that could potentially damage the patient-provider relationship. Some HCPs interviewed had received communication training, but for most, this training was not specific to vaccination. Although general communication skills were transferable to vaccine conversations, most HCPs welcomed specific training and informational resources to support countering patients’ misconceptions or misinformation about vaccines.

**Conclusions:**

HCPs would benefit from training tailored to address vaccine communication with patients, and this should be part of a systemic approach that also provides time and space to have effective vaccine conversations.

**Supplementary Information:**

The online version contains supplementary material available at 10.1186/s12875-024-02509-y.

## Background

Healthcare professionals (HCPs) fulfil an important role not just in vaccinating the population, but also in informing patients and caregivers about the benefits and risks of recommended vaccines and addressing their concerns about vaccination. Effective communication is critical to increase patients’ knowledge and acceptance of vaccine offers. Most people view HCPs as a major and trusted source of vaccine information [8, 13, 35], who can offer reassurance and positively impact vaccine decision-making [25, 36, 37]. HCPs thus need to be adequately prepared for their communication role and the challenges that may arise as part of it, for example, when speaking with hesitant patients [11, 47]. Vaccine hesitancy that HCPs may encounter covers a continuum that includes “delay in acceptance or refusal of vaccination despite availability of vaccination services” [33].

There are several challenges in mobilising HCPs to proactively recommend vaccination. First, HCPs’ own attitudes towards vaccines can impact whether they recommend vaccines to patients [3, 18, 51, 52]. Even if HCPs are themselves in favour of vaccination, their personal attitudes may conflict with their desire to enable patient autonomy [21, 30]. HCPs may be themselves uncertain [47], especially when they need more up-to-date information to reassure themselves of the benefits and costs of newer vaccines [37]. Second, HCPs need relational skills in addition to factual knowledge. These include skills for conveying knowledge in a participatory, non-judgemental manner that displays empathy and maintains the patient-provider relationship [9], and skills at debunking misinformation that patients may believe [44]. Training needs to address vaccine communication challenges have previously been identified in primary and secondary care settings [1, 29, 31, 41, 48, 49].

Several training interventions to promote tailored, participatory communication in vaccination-related consultations have been developed and reviewed in recent years [27], for example, Motivational Interviewing [14], the announcement approach [7], and the Empathetic Refutational Interview [20]. Yet these interventions have not been systematically rolled out in HCP training programmes [48]. This could be because communication interventions are not always converted into accessible training or resources for HCPs [22]. It could also be because communication skills are not often prioritised in medical training, except on very sensitive aspects of medicine such as announcing a diagnosis of cancer [39]. Indeed, vaccination training has typically focused on vaccine knowledge and practical skills required to deliver vaccines [43].

In addition, communications training tended to target the informational content of the communication (e.g., risks and benefits of vaccines that one should tell the patient), rather than the style (e.g., using empathetic language and tone to deliver that information; [24, 38]). There is a research gap regarding HCPs’ training needs around identifying and addressing patients’ main barriers to vaccination [24], which this qualitative study addresses. Our objective was to explore HCPs’ experiences and perceptions of the vaccine communication challenges they faced, what training they had received to prepare them for these challenges, and what training needs remained. The findings reported here are of relevance to public health authorities as they can provide a better understanding of the needs of the vaccination workforce to support staff with vaccination roles.

## Method

The research received approval from the School of Psychological Science Research Ethics Committee of the University of Bristol (reference: 119594) and Ethical Committee of Aix-Marseille Université (reference: 2022-10-20-007). The research was conducted in accordance with the ethical principles under the Declaration of Helsinki. All participants gave informed consent prior to participation.

### Setting

We focused on HCPs in England and France who hold vaccination roles (i.e., including at least one of the following tasks: recommending, prescribing, discussing, or delivering vaccination) to explore training provision and experiences under two different health systems. In England, vaccination involves a range of professionals (including, for example, nurses, midwives, pharmacists) in prescription, recommendation, discussion, and delivery of vaccination [45]. In France, vaccination has been mainly carried out by General Practitioners (GPs), but in the last decade, the authorities gradually extended the role of prescribing and delivering vaccination to other health professionals who have undertaken advanced studies (e.g., nurses, pharmacists, midwives; [26, 46]).

In England, the national vaccine training syllabus recommends communicating with patients, but this is only taken as guidance and need not be offered in practice [38]. In France, vaccine communication is a compulsory aspect of physicians’ medical training and clinical examinations, but how the training is delivered is left up to each medical school [23]. Nurses in France undergo compulsory training that includes 4–5 h of vaccination theory during three years of medical studies, with practical training on recommending and administering vaccines completed during medical placements [4].

### Recruitment of participants

HCPs whose roles involved vaccination (i.e., at least one of the following: prescribing, recommending, discussing, and/or delivering vaccination) were invited to take part, with recruitment aiming for a range of ages, genders, professions, and settings (in England, different geographical regions; in France, rural or urban settings). Participants were offered compensation for their time (£20 shopping vouchers in England; €50 in France).

In England, participants were recruited via email from a mailing list of 104 previous participants (response rate = 22%) who had agreed to be contacted for interviews after completing an earlier study on vaccination attitudes and behaviours [17, 18]. In France, participants were recruited by contacting General Practitioners (GPs) through publicly available list-serves, asking participants to share invitations with colleagues, and requesting the Regional Union of Healthcare Professionals for Nurses (UPRS Infirmières) to send out invitations. Seventy invitations were sent to HCPs in southeastern France (response rate = 25%). Recruitment in both countries continued until the researchers agreed that an appropriate range of healthcare professionals were represented and reasonable data saturation to meet the main research objectives had been achieved [42].

### Consent and interview procedures

Prior to the interview, participants were sent an information sheet and consent form electronically. Participants returned the signed consent form electronically or verbal consent was obtained during the interview.

All interviews were held over video- or tele-conferencing and audio-recorded. Semi-structured interviews were conducted in English and French by experienced qualitative researchers with the appropriate language skills (AG, ECA, HF, DH). Interviews were planned to take between 30–60 min, following a topic guide that specified key question areas and prompts for interviewers (see Supplemental Material).

### Data analysis

All interview recordings were transcribed verbatim. French transcripts were translated into English by an English-speaking researcher fluent in French (AG) with the assistance of two native French speakers (PV, EB) where necessary. We followed familiarisation steps in thematic analysis protocols [6]: at least one researcher from each team (AG, ECA, DH) read all transcripts from their country to understand the data and identify potential themes in the transcripts. Based on this preliminary analysis, and guided by the framework method [15], the research team created a framework to systematically analyse the transcripts with respect to four domains relevant to understanding HCPs’ vaccine communication training needs and provision (Table 1, column 1). Two researchers (DH, AB) then read and coded all transcripts with regards to the initial framework, with additional themes identified and added after discussion. Coding was completed using NVivo 13 [32]. To ensure consistency in coding, the two researchers first independently coded the same four transcripts (10%) and discussed them to reach consensus. Two rounds of independent coding and review were completed, first to reach consensus on the four domains, and then on the themes identified within the four domains. All coding discrepancies were resolved through discussion and the framework was updated iteratively between rounds of discussion to improve consistency of interpretation. Thereafter, the remainder of transcripts were coded individually by DH and AB, who met regularly throughout the coding process to discuss any uncertainties.

**Table 1:** Summary of themes from four domains related to HCPs’ vaccine communication and training needs

Domain	Themes	Summary of analysis
1. HCPs’ approach to vaccine conversations	A. HCPs’ perception of their communication role.	Vaccine communication is part of HCPs’ role. HCPs felt that: • They needed to provide information so patients could make informed vaccination decisions (e.g., “we need to first simply inform [about] this vaccine”). • They communicated about vaccines within their patient-facing roles but some also discussed vaccination with family, friends, and colleagues (e.g., “[my family] asked me often…to explain [vaccination] to them”).
	B. Informational content of the conversation.	In conversations, HCPs described: • Using scientific and medical information to inform and correct patients’ misconceptions (e.g., “with arguments that have a bit of scientific proof”). • Explaining to patients why vaccination was beneficial to individuals and for the collective (e.g., “to protect baby from infections”). • Sharing personal experiences and anecdotes (e.g., "I tell them that I was sick") when this information was deemed relevant.
	C. Communication styles HCPs use in vaccine conversations.	• Many HCPs were comfortable initiating conversations about vaccination (e.g., “actively [promoting] vaccinations to everyone”) and encouraging their patients to be vaccinated, but some HCPs felt uncomfortable and would refrain from pursing the subject further if the patient was hesitant. • HCPs highlighted the need to respect patient autonomy, but characterised this in different ways, for example: • Remaining neutral at the start of the conversation and “not push [the patient] in either direction”. • Letting the patient make the ultimate decision but listening and trying “to help them with their choice” • HCPs’ goals in the conversation included providing reassurance, information, and explanations about vaccines. HCPs from England additionally mentioned encouraging patients to look up information for themselves. • Some HCPs had been trained to use communication techniques to empathise with and demonstrate openness to their patients.
	^a^D. HCPs’ perception of the experience of vaccine-related conversations with patients.	• Conversations described as “difficult” and “unpleasant” tended to involve challenges for HCPs in communicating. • Conversations described as “easy” and “comfortable” tended to involve patients listening, good HCP-patient relationships, and the HCP feeling prepared and confident in their communication skills.
2. Challenges HCPs face in vaccine communication	A. Difficulties in addressing misconceptions/misinformation and/or changing patients’ minds.	HCPs faced challenges with patients displaying resistance to vaccines, generally in response to a vaccine recommendation, but also towards vaccination mandates. Challenges included: • “Legitimate” doubts and concerns where HCPs appreciated their patient had “a logical position”. • “Irrational” concerns, where HCPs questioned the reasoning to reject vaccination (e.g., “fear of needles and they were covered in tattoos”). • Misconceptions coming from unreliable sources (e.g., Facebook) or misinterpretations of credible information sources (e.g., patients “[coming] to [their] own conclusion about [a scientific paper]”). • Religious objections and conspiratorial beliefs, especially extreme cases where patients became aggressive and accusatory. • A perception that hesitant patients’ mindsets could be difficult to change, which discouraged HCPs from continuing the conversation. However, some HCPs were willing to engage with this challenge, reflecting that “we hope that having that conversation may make them shift [their fixed mindset]”.
	B. Lack of information, including lack of up-to-date information to give patients.	HCPs had experienced lack of knowledge and uncertainties around responding to concerns about the COVID-19 vaccination, where they felt uncomfortable because: • There was a lack of official information resources particularly at the start of the vaccine roll-out. • There were delays in receiving official government advice. • They had encountered conflicting information. • They had doubts about the necessity of COVID-19 vaccination for some of their patients. Aside from this, some HCPs identified information deficits in producing convincing counterarguments to misconceptions, particularly those motivated by misinformation or conspiracist beliefs.
	C. Needing time and space to have the conversations.	The time taken to respond to patients’ doubts and concerns posed some challenges: • HCPs working in primary care reported having short consultations where they “don’t have the time built into consultations for [discussing vaccination]”. • Some HCPs in England had found effective organisational solutions to make time for vaccine conversations, including creating opportunities for “having chats with patients rather than bringing them in on the fast-paced in/out clinics”.
	D. How communication skills can help in difficult conversations.	HCPs agreed that communication skills were important but developing those skills were challenging and that HCPs who lacked these skills were unable to “give the best experience [to patients]”. HCPs described a variety of communication skills they used to try and reach positive outcomes in challenging conversations, including: • Positive non-verbal communication. • Active listening to clarify patients’ concerns. • Adapting their response to the patients’ needs, especially when patients were ambivalent. • Reminding patient of their autonomy and expressing respect—however, in some cases, this mean the HCP toned down recommendations or did not pursue the conversation further.
	E. Needing to maintain relationships with patients and avoid conflict.	HCPs highlighted trust as an important component that facilitated vaccine conversations and increased patients’ receptivity. Strategies to build and/or maintain trust included: • Providing continuing care and getting to know patients on a personal level. • Communication skills (e.g., “body language, how you talk to them”) • Ensuring a conducive environment for conversations that was “not in a public space” and did not “look like you are superior and you are telling them what to do”. • Avoiding getting into arguments or damage existing relationships be enforcing the HCPs’ own beliefs about vaccines since this was only one aspect of the care relationship. • Backing away from discussing vaccines with unreceptive patients or avoiding giving certain vaccine information.
3. Vaccination-related training and learning among HCPs	A. Existing training coverage (including *how* and *what*).	All but one of the HCPs interviewed described receiving practical training to administer vaccinations: • Most training was around theoretical and practical aspects of vaccine delivery, including procedures and techniques of vaccine delivery, including obtaining informed consent, and information about how vaccination works, contents of different vaccines, and the country’s vaccination schedule. • Only a few HCPs had covered discussions with vaccine-hesitant patients in their training. • HCPs in France described modules completed during initial professional training, while HCPs in England described specific training offered before taking up vaccination roles. • Some HCPs had received training on communicating with patients in the context of other professional roles. A few HCPs in France had received general Motivational Interviewing training at an earlier career stage.
	B. Experiential learning (from peers and work experience).	HCPs described learning that took place without direct instruction, especially those who had been in the profession for decades. This type of learning included: • Professional experience of speaking to patients providing transferable expertise for vaccine conversations. • Learning skills from watching supervisors and colleagues. • Discussing best practice with peers, e.g., in forums with other HCPs. A few colleagues specifically highlighted pharmacists as good colleagues to learn from.
	C. Official information sources consulted.	HCPs described self-directed learning in the form of seeking out information for their own knowledge and to use in patient consultations: • HCPs described “reliable information” as coming from national public health organisations such as the National Health Service in England and the regional health agency in France. • Many HCPs in England explicitly cited the official government resource for vaccination procedures in the UK (the “Green Book"), referring to it as “their bible”. • Some HCPs used the Internet to search for specific information. • Other sources of information included news media, professional bodies, independent organisations, scientific publications, and pharmaceutical companies.
	^a^D. Perception of informational resources.	HCPs had differing views on the usefulness of information they had access to about vaccination: • A few felt information needed to be summarised as they were busy and “do not have the time to look into it” • HCPs in England felt the UK official government resource (“Green Book”) was cited as useful because it was “incredibly well organised” with “the right amount of actual research” and “provided a framework for knowing what I need to talk to [patients] about”. • HCPs felt they lacked lay information to give directly to patients, commenting that leaflets they had been provided had “a lot of information” and “could be a bit more simple probably”. • Some HCPs felt they could find appropriate information for their patients on official websites, where the information was presented “in quite a logical manner without there being too much information overload”.
4. HCPs’ perception of existing communications training	A. Perceptions about content of existing communications training.	HCPs reported on vaccine communication training they had either attended themselves or knew about from colleagues’ experiences: • HCPs in England had attended COVID-19 vaccine training modules that talked about “the things you can say to encourage uptake”. • HCPs in France mentioned that Motivational Interviewing was “something that is new that is now proposed as a training module” for medical interns. • HCPs in both countries mainly described communication training received as part of other roles or as students, rather than specifically for vaccination. They saw many of these communication skills (e.g., “practical situations with complicated patients…about other subjects”) as being transferable. • Communication training often involved role play and practical situations to simulate patient scenarios, direct instructions about what facts to give patients, and discussions among trainees.
	B. Perceptions of different training formats (e.g., face to face, online).	HCPs described a variety of training formats they had experienced, including webinars, seminars and conferences, online modules, and workshops. Different formats had merits and drawbacks: • Online training was more accessible and could be scheduled without requiring travel. • E-learning could be self-directed, with HCPs going through at their own pace or repeating learning content where needed. • Online training was useful if it was interactive (e.g., “lots of videos and animations…and after each section you are tested on it”), but HCPs felt online presentations often “goes in on ear and out the other”. • Face-to-face training might have logistical challenges, but HCPs perceived it as more enjoyable, with more opportunities for interaction and practice meaning “it was better than learning in books”.
	C. Perceived relevance of existing communications training.	HCPs felt communications training was relevant to their vaccination roles: • Some HCPs felt training that targeted communication style and included practical exercises on this was more useful that training that provided informational content. • HCPs who had undergone generic communications training felt this had relevance to vaccination conversations. HCPs in France who had undergone generic Motivational Interviewing training felt they had been able to put that into practice.
	D. Gaps in training (including if this type of training is not offered).	Training gaps identified included: • No coverage of vaccine communication. • Focus on vaccine knowledge but not “how to sit and talk to a patient”. • Lack of training on how to deal with patient misconceptions and vaccine misinformation and how to better target communications to different patients. HCPs wanted vaccine communication training to: • Learn new skills, refresh their knowledge, and increase their confidence. • Help with identifying patients’ motivations for vaccine hesitancy. • Incorporate difficult conversations they might encounter around misconception and misinformation. • Learn how to “stop short of an argument” when discussing vaccination. • Provide conversation templates “as a roadmap to be able to discuss [vaccination] with people”. HCPs felt training providers might assume HCPs already had the requisite communication skills. Some HCPs with longer clinical experience felt they could already communicate well with patients, but many HCPs perceived a skills gap around communication in the workforce, especially as “there are other vaccinators and some nurses who are not [confident with conversations] because they’ve not had the privilege of the years’ experience”.

^a^Indicates themes added to the initial analysis framework after discussion during the coding process

## Results

Between July to November 2022, we interviewed 41 HCPs from England (*n* = 23) and France (*n* = 18). Socio-demographic characteristics can be found in Table 2. Participants were from a range of healthcare professional specialisms that covered the full range of vaccinations included in the immunisation schedule for each country (with nine reporting experience with multiple specialisms). Nearly half (*n* = 18) of participants worked in primary care settings, meaning their roles included vaccinations for multiple age groups as recommended in the immunisation schedule.

**Table 2:** Sociodemographic characteristics of interview participants

	Number (%)		
Characteristics	France (*n* = 18)	England (*n* = 23)	Total (*n* = 41)
Profession			
General practitioner (GP)	8	7	15 (37%)
Nurse	9	15	24 (58%)
Other^a^	1	1	2 (5%)
Sex			
Female	9	17	26 (63%)
Male	9	6	15 (37%)
Age			
≤ 30 years	5	3	8 (20%)
31-49 years	7	7	14 (34%)
≥ 50 years	6	13	19 (46%)
Region (England)			
East of England	-	8	-
South East	-	4	-
London	-	2	-
West Midlands	-	3	-
South West	-	6	-
Region (France)			
Rural	4	-	-
Urban	14	-	-
Professional specialism related to vaccination role and experience			
Primary care (general medicine)	9	9	18 (44%)
Secondary care (hospital care)	5	2	7 (17%)
Adolescent vaccination services (e.g., schools)	-	3	3 (7%)
Mass vaccination programme (typically COVID-19)	-	3	3 (7%)
Maternity care	-	2	2 (5%)
Other/no specialism^b^	4	4	8 (20%)

^a^Other = pharmacist (France) and community health worker (England)

^b^Other = mental health service (*n* = 2), sexual health service, and community health (England)

4 participants in France reported no specialism. Nine HCPs reported more than one specialism, but only the main specialism is classified here

Table 1 shows the final themes identified through thematic analysis of the transcripts and a summarised version of the results presented below. These results reflect the key issues that relate to HCPs’ vaccine communication with patients and their unmet training needs, organised by the four domains and their main themes. We illustrate each theme with quotations that concisely represent typical responses of interviewees from both countries.

### HCPs’ approach to vaccine conversations

#### Perception of communication role

All HCPs recognised vaccine communication was part of their role. Many felt that they needed to provide information for patients to make their own informed decisions on vaccination.

*Before taking a position or not, we need to first simply inform that there is this or that vaccine, that certain ones are mandatory, some are recommended, that some are reimbursed, some are not…and then according to the reception of this information, [we have] an advising role.* (*P04, Male, 41, GP (Primary care), France*)

Most HCPs discussed communication in the context of recommending vaccines to patients and answering their questions about vaccines, but several also highlighted that they discussed vaccination outside of their patient-facing roles, for example with family, friends, and colleagues:

*I had members of my family even who were against it…but they asked me often about what I thought and to explain [vaccination] to them.* (*P16, Female, 26, GP (Primary care), France*)

#### Informational content of conversation

HCPs often described using scientific and medical information to inform and correct misconceptions.

*I tried to stay with arguments that have a bit of scientific proof...even looking up in front of them studies that show the decrease in incidence of the disease since vaccination began.* (*P15, Male, 35, GP (Primary care), France*)

HCPs would also explain to patients why vaccination was beneficial from an individual as well as collective standpoint.

*I will talk about the benefits of having the vaccinations to protect baby from infections, pros and cons and that sort of thing with new mums.* (*P28, Female, 42, GP (Primary care), England*)

Some HCPs also mentioned that they would share personal experiences and anecdotes when they felt this information was relevant to encourage their patient to be vaccinated.

*I tell them that I was sick. That I was almost on a ventilator, because they see me as someone who is strong…a solid guy, a doctor. Someone who isn’t fragile.* (*P12, Male, 67, GP (Primary care), France*)

#### Communication style

Many HCPs were comfortable with initiating conversations about vaccination and encouraging their patients to be vaccinated.

*I try to promote actively vaccinations to everyone, every patient, children and adults and I use that in every contact that I have in the surgery.* (*P20, Male, 46, GP (Primary care), England*)

However, some HCPs felt uncomfortable if the patient was hesitant and would refrain from pursuing the subject of vaccines further.

*I didn’t respond [to the patients’ concern], in fact. I knew that the communication was complicated, and so if they asked me questions, I responded, but after, I left them to their beliefs.* (*P11, Female, 41, Nurse (Secondary care), France*)

When speaking with hesitant patients, most HCPs highlighted the need to respect patient autonomy in their vaccination decision. For some, this meant remaining neutral at the beginning of the conversation.

*I try not to push them in either direction, I just give them the information and just say, if you would like to have these vaccines then just make an appointment. (P22, Female, 33, GP (Primary care), England)*

For others, respecting autonomy meant letting the patient make the ultimate decision but still trying to support them with that decision.

*Even if for me, [although] I find [it] a shame to not vaccinate…from the moment that [patients] are aware of the risks…we listen and try to help them with their choice while respecting their wish to not get vaccinated.* (*P08, Male, 42, Nurse (No specialty), France*)

Some HCPs from England reported encouraging patients to look up reliable information for themselves. This approach was not mentioned by HCPs in France, although they discussed similar goals of providing reassurance, information, and explanations about vaccines.

*My approach is to reassure them, show them the information we have got and with pregnant women I’ve found a link to a podcast which I thought they might like to follow up on.* (*P19, Male, 62, Nurse (Mass vaccination programme), England*)

Some HCPs described communication techniques they had been trained to use, for example, counselling skills, how to listen, ways to tailor information, and the use of analogies. The purpose of these techniques was to reflect empathy and openness towards their patients.

*It’s about listening and about hearing what that objection is and then to try and relate it to the current day.* (*P36, Female, 64, Nurse (Maternity care), England*)

#### Perception of vaccine conversation experience

 HCPs described some vaccine conversations as “difficult” and “unpleasant”, in which they faced challenges in vaccine communication detailed in the next section. In contrast, other vaccine conversations were described as “easy” and “comfortable”. In these conversations, patients listened, HCPs had a good relationship with the patient, and HCPs had confidence in their own communication skills and felt prepared for the conversation.

*You think beforehand [of] all the scenarios of what you might be asked. That’s how…in the conversation I didn’t feel challenged. I think [the patient] was quite happy to receive [the information].* (*P27, Female, 52, Nurse (Adolescent vaccination services), England)*

### Challenges in vaccine communication

#### Difficulties in addressing patients’ misconceptions and fixed beliefs

HCPs described various challenges they faced when patients displayed resistance to vaccines. This was generally in response to the HCP’s recommendations, but a few HCPs had also faced challenging patients who arrived for their vaccination upset about vaccination mandates (e.g., for travel or professional purposes).

*There are a lot of people who did [vaccination] really for professional reasons. It is [these] people who would come and be angry.* (*P10*, *Female, 56, Nurse (No specialty), France*)

HCPs described some of the doubts and concerns of their patients as legitimate, but others they considered irrational. For example, HCPs appreciated their patients’ logic for declining vaccination.

*Those [patients] that believe that the data is not sufficiently robust enough and don’t wish to be part of a large experiment until they’ve got longer term data…we’ve peddled that line ourselves with new drugs and new technologies all the time. So, although it’s not a position that I think is the most sensible, it is a logical position.* (*P23, Male, 52, GP (Primary care), England*)

In other instances, HCPs questioned the reasoning that some patients used to reject vaccination.

*[The patients’] reasons were fear of needles and they were covered in tattoos…so there’s some warped perceptions of what they’re prepared to put themselves through or not.* (*P41, Female, 57, Nurse (Secondary care), England*)

HCPs were able to detail some misconceptions patients had about vaccines, which many HCPs identified as coming from unreliable information sources such as social media.

*The paradox is that they have more confidence in Facebook groups than in studies.* (*P01, Male, 25, GP (Primary care), France*)

One HCP reflected that even credible information sources could be misinterpreted by people without the right expertise.

*Without the real expert understanding and knowledge and everything that happened behind that, in some ways [the sources] are more dangerous than they are informative at times. … [The patient] read a BMJ [British Medical Journal] paper…She basically found what we’d call credible evidence, but then came to her own conclusion about it.* (*P36, Female, 64, Nurse (Maternity care), England*)

The most difficult experiences cited included patients with religious objections and conspiratorial beliefs. In extreme cases patients became aggressive, accused HCPs of being part of the conspiracy and of wanting to harm children with vaccinations.

*That is also the problem, that they think so much about the conspiracy that you give an argument in favour of vaccination and they envelop you in the conspiracy. (P01, Male, 25, GP (Primary care), France)*

Many HCPs recognised that hesitant patients’ mindsets could be difficult to change. Some HCPs found this resistance to change challenging and were discouraged from continuing vaccine conversations with these patients:

*From the moment I understand that no matter what my response [is], it will not change their way of thinking...I let it go.* (*P02, Female, 52, GP (Primary care), France*)

Others displayed confidence and willingness to engage with patients with such mindsets nonetheless, reflecting that the conversation might still do some good.

*The people of that cohort are usually of a fixed mindset and it’s quite difficult to shift that, and we hope that having that conversation may make them shift it.* (*P21, Male, 33, GP (Primary care), England*)

#### Lack of information to give patients

HCPs most commonly discussed lack of knowledge and uncertainties in how to respond to concerns in the context of COVID-19 vaccination programmes. Particularly at the start of the vaccine roll-out, HCPs struggled with the lack of official information resources to support evidence-based conversations with patients and delays in receiving official government advice. HCPs had encountered conflicting information that contributed to their uncertainties in responding to patients and felt that reliable information was often obscured amidst large amounts of false information on the Internet.

*What made me uncomfortable was also that I didn’t have enough information… [patients] would say “okay, tell me what are the side effects, there are women who aren’t able to have children any more” and I was uncomfortable because I didn’t really know how to respond*. *(P09, Female, 57, Nurse (Secondary care), France*)

Two HCPs from France also had doubts about the necessity of COVID-19 vaccination for some of their patients.

*As a citizen, I do not really agree with vaccinating the youngest [against COVID-19], for example.* (*P14, Male, 52, Nurse (Primary care), France*)

A more general information deficit for some HCPs was the lack of convincing counterarguments for patients’ misconceptions, particularly when these concerns were motivated by misinformation or conspiracist beliefs and patients did not believe the factual information the HCP had provided.

*She was saying how she was reading conspiracy theories online. I didn’t really know how to address that one, but I was just trying to say to her, “It is effective, it does go through all these clinical trials, so it is very much safe.”* (*P32, Female, 29, Nurse (Mental health services), England*)

#### Needing time and space for vaccine conversations

Some HCPs highlighted the substantial time it took to respond to patients’ doubts and concerns about vaccines. This was especially challenging for HCPs working in primary care. Often they reported having short consultations where the principal focus was not vaccination, leaving limited time to dispel vaccine misconceptions or engage in a convincing discussion with hesitant patients.

*I don’t have the time built into my consultations for it…if they’re coming for something else, to then add that on to the consultation that’s another 10 minutes and I’ve got another patient waiting so it’s quite tricky.* (*P28, Female, 42, GP (Primary care), England*)

Some HCPs in England shared experiences where their organisations had implemented effective solutions to make time for vaccine conversations. These generally involved creating opportunities for patients to speak with a medical professional, for example:

…*having chats with patients rather than bringing them in on the fast-paced in/out clinics…being able to have time with patients provides a more positive reinforcement and outcome for the patients and their experience with having the vaccine.* (*P33, Female, 27, Community health worker (Community health), England*).

#### How communication skills help with difficult conversations

HCPs shared some of their strategies to try and reach positive outcomes when they engaged in challenging vaccine conversations. A variety of communication skills were described, for example positive non-verbal communication and active listening to clarify the patients’ concern and enable the HCP to adapt their response to the patients’ needs.

*It’s about that paraphrasing…so that you understand what the actual concern is before you answer them, because otherwise you’re just assuming what their anxiety or fear is about rather than finding out.* (*P29, Female, 56, Nurse (Mass vaccination programme), England*)

Many HCPs would remind patients of their autonomy and tell the patient they respected the patient’s choice. In some cases, this meant the HCP did not pursue the conversation any further, or would tone down their vaccine recommendations for vaccine hesitant patients whom they thought would react poorly to strong recommendations.

*I know through experience that that doesn’t serve any good to take a strong position that could seem condescending to people who, they themselves are against vaccines.* (*P04, Male, 41, GP (Primary care), France*)

The ability to adapt the conversation to the patient was also described as particularly important when speaking to patients who were ambivalent with regards to accepting vaccination.

*There are things to put in place and things to say and things to not say…to adapt the discussion…to explain to those who are “convincible”…I think there are people for which there are arguments and things can be done to bring them onto the side of vaccination.* (*P15, Male, 35, GP (Primary care), France*)

HCPs agreed that despite their importance, developing effective communications skills was challenging. Some HCPs mentioned professional experiences and training that helped develop these skills, but they recognised that not all their colleagues had the skills to communicate well.

*Some vaccinators can’t do that [communicate well] so I don’t think they’ve given the best experience [to patients].* (*P38, Female, 66, Nurse (Adolescent vaccination services), England*)

#### A need to maintain patient-provider relationships

Many HCPs highlighted trust as an important component that facilitated vaccine conversations with patients by increasing patients’ receptivity to the HCP’s vaccine recommendations. Most HCPs raising this theme provided continuing care in a broader area (e.g., primary care, mental and sexual health services). For them, getting to know patients on a personal level was one way to develop this trusted relationship.

*I think it helps if you have got a relationship with that patient already. If they know you and trust you, if you say things to them, they’re much more likely to hear them.* (*P39, Female, 51, GP (Primary care), England*)

Some HCPs described how communication skills helped to build trust, for example by ensuring the environment was conducive for the conversations.

*It is how you use your body language, how you talk to them and it is just general interaction. … We have got an area where we can actually take them [we talk] one to one and not in a public space. I’m coming around to chat to them face to face, to break down any barriers because when you’ve been sitting behind a table it looks like you are superior and you are telling them what to do.* (*P19, Male, 62, Nurse (Mass vaccination programme), England*)

Although HCPs believed that trust was helpful for effective vaccine communication, it could in some circumstances also be counterproductive.

*It is good because they trust us, and so that helps to speak openly, but sometimes what is bad is that because they treat us like family, sometimes they don’t listen to us.* (*P06, Male, 37, Nurse (No specialty), France*)

HCPs’ prioritisation of maintaining a trusted relationship could also lead them to avoid giving certain information or back away from discussing vaccines if they sensed patients were unreceptive or that the conversation would take too much time. HCPs mentioned not wanting to get into arguments or damage existing relationships by enforcing their own beliefs about vaccines, particularly as vaccination was only one aspect of their care relationship with their patients.

*You can only try so far and then you can tell if you’re starting to alienate them and you’re affecting your relationship with them so I think you just have to respect their decision…you do have to back off.* (*P30, Female, 65, Nurse (Sexual health services), England*)

### Vaccination-related training and learning

#### Existing training coverage

 All but one of the HCPs interviewed described receiving practical training to administer vaccinations. This focused on procedures and techniques of vaccine delivery, including obtaining informed consent, in addition to information about how vaccination works, the contents of different vaccines, and their country’s vaccination schedule. Many HCPs in France described vaccination-related modules they had completed during their initial professional training, while many HCPs in England mentioned vaccine-specific training that was available before taking up vaccination roles. Most training was on vaccine theory and practical aspects; only a few HCPs mentioned their courses tried to address discussions with vaccine-hesitant patients.

*It was just theory when we talked about [vaccines], when we were in school. That was several years ago and now, [there is] nothing in particular for vaccination.* (*P17, Female, 47, Nurse (Secondary care), France*)

Some HCPs described training they had received around communicating with patients, mostly in the context of their other professional roles and not specific to vaccines.

*I actually did the Diploma in Child Health…so I’ve done that sort of communication skills and the knowledge that you need during [that] training.* (*P23, Male, 52, GP (Primary care), England*).

In France, a few HCPs highlighted motivational interviewing training that was available at an early career stage (though it was not only targeted at vaccination).

*During [my] internship, I followed a training about motivational interviewing…that can also be used, for example, for tobacco. (P03, Female, 28, GP (Primary care), France)*

#### Experiential learning

HCPs mentioned that learning took place without direct instruction during their professional training. Most of these HCPs had been in the profession for decades and they felt the many patients they had spoken to over the years helped them to gain transferable expertise with patient conversations.

*I was a surgical nurse for quite a number of years…we would have to impart bad news…so actually you learn those communication skills.* (*P31, Female, 50, Nurse (Adolescent vaccination services), England*)

Professional peers were also a source of experiential learning for HCPs, as HCPs picked up skills from watching their supervisors and colleagues.

*I think the team in the whole are quite skilled at communicating…and then the new staff coming through hear those conversations all the time so they learn from it.* (*P37, Female, 51, GP (Primary care), England*)

HCPs also described useful opportunities for discussing best practice with peers, for example in forums with other HCPs. Pharmacists were highlighted as good colleagues to learn from as they had good knowledge of vaccines and, specifically in France, often spoke to patients.

*We were in contact with pharmacists because they were our intermediaries [with patients]…the pharmacist would say, “oh well if you have all of these doses do it this way.” (P07, Male, 57, Nurse (Secondary care), France)*

#### Official sources of information consulted

HCPs tended to seek out information on vaccines for their own knowledge and to use in consultation with patients, as a form of self-directed learning. Most explicitly mentioned using “reliable information” coming from national public health organisations (e.g., in England, the National Health Service “NHS”; in France, the national health insurance fund website “AMELI”, regional health agency “ARS”, and expert health authority that recommends vaccines “HAS”).

*We made it very clear that we would only access information from two places [the government and the NHS], and if there was any wealth of information elsewhere, we would just simply acknowledge it but we wouldn’t use it ourselves in sharing to others.”* (*P21, Male, 33, GP (Primary care), England*)

In England, many HCPs explicitly cited the Green Book (the official government resource for vaccination procedures in the UK), with most describing it as “their bible”. Some HCPs also used the Internet to search for specific information; others discussed information they got from news media, professional bodies (e.g., French Society of Infectious Diseases), independent organisations (e.g., Oxford Vaccine Group), scientific publications, and pharmaceutical companies.

#### Perception of informational resources

HCPs had differing views on the usefulness of information they had access to about vaccination. A few felt they were too busy to look through the information and that it would be helpful if it could be summarised.

*We receive the Revue du Praticien* 1* at the practice but I admit I absolutely do not have the time to look into it.* (*P02, Female, 52, GP (Primary care), France*)

The Green Book (the resource for UK vaccination) was specifically highlighted as a useful resource because it was “*incredibly well organised and contains the right amount of actual research and also stating the facts simply but not too much complex detail that it’s not easy to understand*” (*P35, Female, 25, Nurse (Primary care), England*) and provided “*a framework for knowing what I need to talk to [patients] about*” (*P23, Male, 52, GP (Primary care), England*).

With regards to information resources to share with patients, HCPs felt they lacked lay information that they could give directly to patients. Some HCPs felt that the official vaccine information leaflets that were provided for patients were unsuitable.

*I think the leaflets that are there trying to explain to the patients in layman’s language about the vaccine…I’ve never liked them … I think it’s a bit too much sometimes…it is a lot of information—I’m not sure who is going to read that. I think it can be a bit more simple probably.* (*P20, Male, 46, GP (Primary care), England*)

However, others mentioned that they could find appropriate information for their patients on official websites such as that of the NHS.

*[The information was] broken down into easy to digest chunks...I think it presents it in quite a logical manner without there being too much information overload.* (*P22, Female, 33, GP (Primary care), England*)

### HCPs’ perception of vaccine communication training

#### Content of existing communications training

Some HCPs gave details of vaccine communication training they had either attended or knew about from colleagues. For example, one described a COVID-19 vaccine training module where “*there was a brief aspect to the module where it goes on to say when people are not sure about having the vaccine, these are the things that you can say to encourage uptake*” (*P21, Male, 33, GP (Primary care), England*). Another mentioned that motivational interviewing for vaccine conversations was “*something that is new that is now proposed as a training module when we are interns*” (*P41, Male, 41, GP (Primary care), France*)—although it should be noted that such vaccine-specific communications training for medical interns is still not compulsory in the French system.

More commonly, HCPs described communication training they had received as students or in the context of other professional roles rather than as preparation for their vaccination duties. This training covered skills that were transferable such as how to involve patients in their own care, how to convey bad news to patients, and how to navigate tricky conversations with people.

*We had some sessions of practical situations with complicated patients. But…it was about other subjects, like about antibiotics for example, or announcing a serious disease.* (*P16, Female, 26, GP (Primary care), France*)

HCPs described the use of role plays and practical situations in their general communication training to simulate patient scenarios, direct instructions about what facts to give patients, and sessions where trainees had discussions and sharing of experiences.

#### Formats of existing training

HCPs described a variety of training formats they had experienced, including webinars, seminars and conferences, online modules, and workshops. HCPs acknowledged that different training formats had their merits and drawbacks. Online training was generally seen as more accessible, allowing HCPs to schedule it into their day without needing to travel to a training location. One advantage of online training (specifically, “e-learning”) was its ability to be self-directed so individuals did not need to be present at a fixed time and could go through materials at their own pace or repeat learning content. In some cases, it could also be designed to be interactive.

*I quite like it when it's smaller blocks, and when it has lots of pictures. I like lots of videos and animations and things like that…I learn much better that way.…I quite like that after each section you are tested as well on it, because that helps to cement it in place.* (*P40, Female, 51, Nurse (Maternity care), England*)

However, when this type of training was limited to an online presentation, some HCPs questioned its utility.

*Half the time it's going in one ear and out the other, and by the time [one goes] back to work it's like, “Well, what did I learn?”* (*P33, Female, 27, Community health worker (Community health), England*)

Although face-to-face training presented logistical challenges, many HCPs felt that it was more enjoyable and provided more opportunities for interaction and practice through various exercises.

*I think it was good to have a training with role playing and the trainers who explained things well, it was better than learning in books.* (*P05, Female, 27, GP (Primary care), France*)

#### Relevance of existing communications training

Most HCPs who had experienced training related to vaccine communication felt that it was relevant and helpful to their roles and they had subsequently put it into practice.

*I think educating yourself, learning and updating yourself with the latest information, that really gives you the confidence, because you can then impart that information to the patients.* (*P40, Female, 51, Nurse (Maternity care), England*)

Some HCPs felt that it was more useful to target the communication style (e.g., how to approach patients) and include practical exercises, as opposed to just providing informational content (e.g., what to say). These HCPs tended to be younger (aged < 30 years).

*It's always been, “This is the spiel I give them but the best thing you can do is have a read of the leaflet, and then figure out on your own.” When you hear things like that you think, I don't quite like the feel of that.* (*P33, Female, 27, Community health worker (Community health), England*)

HCPs in France who had undergone Motivational Interviewing training (for patient communication in general) described how they had put that training into practice.

*I think that [it] helps me sometimes when I don’t have arguments or I feel that the patient is a bit upset, I try to use the basics of the motivational interview to get back on track. And that works pretty well.* (*P05, Female, 27, GP (Primary care), France*)

HCPs who had described communications training in the context of other roles also felt that training had relevance to their vaccination conversations.

*You want to be able to have an engaging conversation and make sure people walk away from it feeling positive. So, having done that training before about managing challenging conversations has definitely been helpful in my role as a vaccinator.* (*P32, Female, 29, Nurse (Mental health services), England*)

#### Gaps in vaccine communication training

The main training gap identified by the majority of HCPs was that vaccine communication was often not covered.

*Never in our training as vaccinators did they say, “This is how you address this, if a person says this,” so [vaccine communication] was just something that I was just a bit unsure about.* (*P32, Female, 29, Nurse (Mental health services), England*)

HCPs felt that their existing training focused on vaccine knowledge but “*they don’t tell you how to sit and talk to a patient*” (*P26, Male, 65, Nurse (Mass vaccination programme), England*). HCPs expressed the desire to have such training, so they could learn new things, refresh their knowledge, and increase their confidence.

*You never know what's going to be said to you and that’s what makes the nervousness, that anxiety around those conversations. For me [what is needed] would be more knowledge, more training, because I think the more that we've got the more we can talk through that and feel that we’re giving great answers with that. (P27, Female, 52, Nurse (Adolescent health services), England)*

However, there was a perception that training providers assumed HCPs would already have these skills.

*All they deal with is the medical thing like when [vaccination is] needed, why it’s needed.… [Communication is] something you just either know how to do or you don’t, they don’t tackle it in training at all.* (*P24, Female, 47, GP (Primary care), England*)

In a few cases, HCPs felt they could communicate effectively due to their professional backgrounds and experience, so training was not necessary for them.

*Being a nurse for 32 years I'm not sure I needed the “how to communicate something” with a patient.* (*P31, Female, 50, Nurse (Adolescent health services), England*)

However, even though HCPs, especially those aged over 50 years, might have built up skills through experience, they reflected that vaccine communication training would still be useful, especially for junior colleagues who did not yet have this experience. Some acknowledged that there was a skills gap in the vaccination workforce when it came to communication.

*It’s not difficult to train somebody to give an injection. What is difficult is you need somebody with the personality to put people at ease. … I do feel confident having difficult conversations because I’ve been trained to do it. I’m sure there are other vaccinators and some nurses who are not because they’ve not had the privilege of the years’ experience that I’ve got. (P38, Female, 66, Nurse (Adolescent health services), England)*

Some HCPs felt there were gaps even in existing vaccine-specific communications training, particularly around dealing with patient misconceptions and vaccine misinformation and how to better target communications to different patients. For example, HCPs wished to know how to identify patients’ motivations for vaccine hesitancy.

*What would help a lot is learning to identify…[the] nuanced side of patients…Once we know who we are talking to, which personality we are talking to, we can use this or that argument [for vaccination].* (*P18, Male, 30, Pharmacist (Secondary care), France*)

HCPs also felt that there were gaps in practical skills and tools offered, for example they wanted training that incorporated the difficult conversations they might encounter around patients’ vaccine misconceptions or misinformation so they could learn “*when to stop short of an argument*” (*P24, Female, 47, GP (Primary care), England*), or have conversation templates to use as “*a roadmap to be able to discuss with people*” (P*12, Male, 67, GP (Primary care), France*).

## Discussion

The objective of the study was to provide insight into HCPs’ experiences and perspective of vaccine communication and the associated training they had received. In semi-structured interviews with HCPs in England and France, we explored how HCPs approached vaccine conversations with patients, the challenges they had in these conversations, what training they had received to support those conversations, their perception of that training and their unmet training needs. We discuss our key findings here with a view to providing public health authorities a better understanding of the training needs of the vaccination workforce to prepare them for challenging vaccine conversations.

The main approaches to and challenges with vaccine conversations were broadly similar in both countries. HCPs often had conversations with patients to provide vaccine information, which required effective communications skills. However, HCPs were not always supported with sufficient time, informational resources, and skills to confidently navigate difficult conversations with hesitant patients without risking damage to the wider patient-provider relationship that they wished to prioritise. This is consistent with other studies conducted in Ireland, the Netherlands, and Australia, which found that HCPs preferred to avoid conflict to maintain their rapport with patients [1, 30, 34, 40].

The HCPs we interviewed had received training in delivering vaccinations, with provision differing between countries. HCPs in France mostly recalled vaccine-related training occurring in earlier stages of their career, whereas HCPs in England largely described ongoing training to update their skills and knowledge. In both countries, training mostly covered practical skills and vaccine-specific knowledge, which is in line with existing literature on vaccine training provision (e.g., [23, 24, 38]). The informational resources HCPs consulted to increase their vaccine knowledge also targeted the content of vaccine conversations rather than communication style.

HCPs who had attended vaccine communication training felt it was useful for dealing with communication challenges. However, the majority of HCPs we interviewed in both countries reported that they had not received such training. This was the case for both nurses and GPs. This may seem surprising since both countries include vaccine communication as a core competency: within the national standard for immunisation training in England and within physicians’ compulsory medical training in France. However, England’s national standard is not mandatory [38] and while medical students in France are examined on vaccine communication, it does not necessarily mean medical schools have prioritised this aspect of their training [23]. Indeed, previous research found that French medical students felt underprepared for questions about vaccination from patients [23]. In addition, none of the nurses interviewed in France reported receiving vaccine communication training, whereas in England, several of the nurses we interviewed had received such training.

HCPs commonly described accumulating communication skills through their clinical role, or applying communication training (including on motivational interviewing) that they received elsewhere to vaccination contexts. HCPs also picked up skills informally from their colleagues, which suggests that training some HCPs in a team could benefit the whole team.

One specific training need highlighted was to provide help finding convincing counter-arguments to patients’ misconceptions. This area was previously identified as needing more research attention [24], and we find that there is indeed a gap in training provision here. HCPs would benefit from training that targets why patients hold vaccine misconceptions and what strategies HCPs could use to successfully correct these. This training gap is especially concerning as many misconceptions held by patients stem from misinformation [2, 16, 28]. HCPs in our sample described cases where it was difficult to challenge information from misleading sources, which patients had encountered and believed.

In line with past research [1], HCPs mostly agreed that vaccine communication training would be useful for them and for their colleagues, especially junior HCPs who could benefit from acquiring relevant communication skills earlier in their careers. Interestingly, the HCPs we interviewed who discussed how communication-focused training would be beneficial tended to be younger (aged < 30 years) and likely closer to their formal academic training, implying that this area was for them still lacking. Thus, it seems promising that HCPs in France mentioned the offer of motivational interviewing training for medical students and interns, though it was not universally available in medical schools across the country nor to nurses. Making this type of communication training specific to vaccination would also be beneficial, as previous work has shown that training medical interns to apply motivational interviewing to vaccination conversations improved their self-efficacy [17], which can translate into more frequent vaccine recommendation behaviour among HCPs [18].

HCPs’ preferences for training delivery reflect a tension between convenience (online training was easily accessible amidst a busy schedule) and enjoyability (face-to-face training was perceived as more engaging and an opportunity to learn through interaction with colleagues). Recent innovations in training delivery—in part accelerated by the need for diverse training media during the COVID-19 pandemic—have provided a range of formats and resources that could be harnessed widely to balance HCPs’ competing needs [5]. This would be especially helpful for HCPs who have difficulty finding suitable times to attend or travel to organised training. Having a range of online and offline resources for learning as groups or individuals, taught or self-directed, would provide flexibility for HCPs to engage in training as part of their continuing professional development. Crucially, regardless of format, training should be interactive to support HCPs’ learning.

Within communication training, specific needs targeting the vaccination context should be addressed. HCPs with more clinical experience or who had attended other forms of communication training expressed confidence with general communication principles, such as listening skills and demonstrating empathy. Some of them described applying these skills successfully in discussions about vaccination, but we do not know how effectively all HCPs can transfer these skills to different contexts. Training in generic communication skills could be more useful for HCPs earlier in their career, when they have not had the opportunity yet to learn from experience. Other specific and complementary skills, such as how to develop convincing responses to correct patient misconceptions about vaccines while preserving the patient-provider relationship, requires greater tailoring of informational resources [9] and sensitivity to the psychological motivations of the patient [14, 20]. As our interviewees described, HCPs may have a tendency to preserve relationships with their patients, sometimes at the cost of vaccine promotion. Most HCPs, regardless of their clinical expertise, are likely to benefit from developing confidence and skills tailored to handle these types of conversations, and this could increase their self-efficacy and likelihood of recommending vaccines [18]. Moreover, vaccine-specific communication training may help to combat counterproductive communication habits such as giving information before listening to the patient [19] or dismissing hesitant patients [10]. Incorporating tailored vaccine communication into HCPs’ training, for example to identify the motivations of patients and align communications with those motivations [20], would also be in line with the WHO’s recommendation to tailor immunisation programmes to understand the perspectives of populations where vaccine coverage is low [12].

Although our main focus was on HCPs’ communicative capability and training provision around this, many of the HCPs we interviewed also brought up systemic issues around when and where vaccine conversations take place, and how this wider structure of the vaccination environment can impact HCPs’ ability to speak with patients. Communication-specific training will not overcome logistical challenges such as a lack of time or dedicated space for a conversation to happen, nor the growing shortage of HCPs that impacts on the care they can provide to patients [50]. It is important to create these conversational opportunities within the healthcare environment. However, this must go hand in hand with preparing HCPs with competencies to engage in effective vaccine communication. Many of the HCPs we interviewed expressed high motivation to speak to patients about vaccines, but they and other colleagues who might be more wary could also increase their motivation to do so with more confidence in their abilities and confidence that patients would receive the conversation well.

### Limitations

Our research offers qualitative insights about how HCPs experience vaccine conversations and training provision around this aspect of their job. However, these in-depth interviews are limited to small samples from each country, so we cannot comment on how prevalent these experiences are among HCPs. Although we recruited a diverse sample in relation to professional roles, regions, genders, and ages, our findings are still limited to a predominantly pro-vaccination sample. The opinions of HCPs who are themselves vaccine hesitant have not been represented as part of this study. Finally, our study only focused on the views of HCPs and did not include patients. We are therefore unable to assess whether HCPs’ perceptions of effective communication match patients’ perceived needs.

## Conclusion

HCPs perceived overcoming vaccine hesitancy through effective conversations as part of their professional role but identified numerous challenges to carrying out their communicative function. HCPs’ training could be improved to teach various specific and complementary skills, such as how to address vaccine misinformation while also communicating in a way that preserves a trustful relationship with patients. Improving such skills may improve HCPs’ motivation to engage in conversations with vaccine-hesitant patients. Training will only be effective when embedded in a supportive system and when its impact is duly evaluated. It should be implemented as part of a systemic approach that provides HCPs with skills, confidence, and logistical support to carry out their vaccine communication roles.

### Supplementary Information

Supplementary Material 1.

## Data Availability

Materials used in the study are provided as Supplementary Material to this article. Qualitative interview transcripts may be requested from the corresponding author.

## Footnotes

1 The *Revue du Praticien* (in English: Practitioner’s Review) is a generalist medical journal.
