# Supplementary material for: Healthcare professionals’ perceptions of challenges in vaccine communication and training needs: a qualitative study
Source: BMC Prim Care. 2024 Jul 20;25:264. doi: 10.1186/s12875-024-02509-y (PMC11265004; doi:10.1186/s12875-024-02509-y)
Supplement: Supplementary file 1 — Supplementary Material 1. [file 12875_2024_2509_MOESM1_ESM.docx]

**Supplemental Material**

**1. Topic Guide for Semi-Structure Qualitative Interviews with Healthcare Professionals**

**SECTION 1: Experiences**

What we’re trying to find out as part of this study is from healthcare professionals, what their experiences are of talking about vaccinations with patients.

1. I’d like to talk to you about your experiences with vaccination. Could you share with me the ways you are involved with vaccination in your work?
2. What do you consider your role to be in discussing vaccinations with patients?

***Patient interactions***

1. Could you tell me about the last interaction you had with a patient where you discussed their doubts and/or concerns about vaccination?
   - How was the subject of vaccines brought up?
   - Can you describe the doubts raised by this patient?
   - How did you respond to these doubts?
   - How easy or difficult was it to continue this interaction with this patient?
   - How did you feel during this interaction? How did this patient affect you?
   - How is this this experience different from the discussions you have with other patients regarding their concerns about vaccination?
2. In general, what stops you from discussing vaccines with patients?
   - Is there anything that could help you feel more confident and supported to have these conversations?
   - Where do your patients say they get their information from concerning vaccines?
   - What tools/resources are available to help you during these conversations with patients?
   - What resources would you like to have available to you?
3. Can you tell me about the last interaction you had with a patient where you felt confident in the communication between you and your patient?

- What do you think made you feel confident during this interaction?

1. Can you tell me about experiences with patients related to non-Covid vaccinations? (if they have only mentioned Covid vaccination)

***Training & information-gathering***

1. Can you describe to me what courses/trainings you have received related to vaccination? (prompts: mandatory, face-to-face/on-line, external or internal providers)
   - What did you like about it?
   - What did you not like about it?
   - What would be the most important thing to change?
   - To what extent do you feel that communication with patients related to vaccinations was addressed during this training?
   - To what extent did you feel prepared to interact with and discuss vaccination with patients after this training?
   - What update/repeat training has been available to you / have you taken?
   - Does your organization give you adequate time and compensation for this training?
2. [If they did not receive any courses/training:]

- Do you think you should receive vaccination-related training?
- What sort of training do you think would be helpful for your role?

1. Where do you usually seek information about vaccines from?
   - Does the source depend on the vaccine?
   - How reliable do you consider these sources of information?
   - To what extent do you feel informed about vaccines?
   - How could you feel better-informed?

**SECTION 2: Covid-19 and vaccination**

a) How has the pandemic changed how you consult with patients?

- - Including:
    1. how appointments are delivered (e.g. online consultations, telephone)
    2. the way you discuss vaccinations with patients?
    3. how you interact with patients?
    4. how patients interact with you?

**SECTION 3: General Questions**

1. Confirm with participant their socio-demographic and professional characteristics
2. Do you have any additional thoughts/questions/concerns that you would like to add before we conclude?

**Original French versions of translated quotations reported in the manuscript**

| Interviewee | Translation (English) | Original French |
| --- | --- | --- |
| P04, Male, 41, GP | Before taking a position or not, we need to first simply inform that there is this or that vaccine, that certain ones are mandatory, some are recommended, that some are reimbursed, some are not…and then according to the reception of this information, [we have] an advising role. | Avant même de prendre position ou pas, nous devons simplement informer, s’il existe tel ou tel vaccin, que certains sont obligatoires, certains sont conseillés, certains sont remboursés, certains ne le sont pas… et ensuite en fonction de la réception de ces informations, [nous avons] un rôle de conseil. |
| P16, Female, 26, GP | I had members of my family even who were against it…but they asked me often about what I thought and to explain [vaccination] to them. | J’ai des membres de ma famille même qui étaient contre… mais ils m’ont beaucoup demandé ce que je pensais et de leur expliquer la vaccination. |
| P15, Male, 35, GP | I tried to stay with arguments that have a bit of scientific proof...even looking up in front of them studies that show the decrease in incidence of the disease since vaccination began. | J’essayais de rester sur des arguments qui ont un peu de preuves scientifiques…limite en recherchant devant eux des études qui montrent la diminution de l’incidence des maladies depuis la vaccination. |
| P12, Male, 67, GP | I tell them that I was sick. That I was almost on a ventilator, because they see me as someone who is strong…a solid guy, a doctor. Someone who isn’t fragile. | Je leur dis que j’ai été malade. Que j’ai été à deux doigts de la réanimation, parce qu’ils me voyaient mes patients comme quelqu’un de costaud… Un type solide, un médecin. Quelqu’un qui n’est pas fragile. |
| P11, Female, 41, Nurse | I didn’t respond [to the patients’ concern], in fact. I knew that the communication was complicated, and so if they asked me questions, I responded, but after, I left them to their beliefs. | Je répondais pas en fait parce je savais que la communication était compliquée, et donc si ils me posaient des questions, je répondais mais après je les laissais dans leurs croyances. |
| P08, Male, 42, Nurse | Even if for me, [although] I find [it] a shame to not vaccinate…from the moment that [patients] are aware of the risks…we listen and try to help them with their choice while respecting their wish to not get vaccinated. | Même si pour moi, je trouve ça dommage de ne pas faire la vaccination… du moment où ils [les patients] ont conscience du risque qu’ils encourent… on écoute et on essaye de les faire avancer dans leur choix mais en respectant leur volonté de pas se faire vacciner. |
| P10, Female, 56, Nurse | There are a lot of people who did it [vaccination] really for professional reasons. It is [these] people who would come and be angry. | Il y avait beaucoup de gens qui l’ont fait [la vaccination] vraiment par contrainte professionnelle. C’est les gens qui arrivaient en colère. |
| P01, Male, 25, GP | The paradox is that they have more confidence in Facebook groups than in studies. | Le paradoxe c’est qu’ils [les patients] font plus confiance à des groupes Facebook qu’à des études. |
| P01, Male, 25, GP | That is also the problem, that they think so much about the conspiracy that you give an argument in favour of vaccination and they envelop you in the conspiracy. | C’est ça aussi le problème, c’est qu’ils [les patients] pensent tellement au complot que dès que tu donnes un argument pour la vaccination, ils t’englobent dans le complot. |
| P02, Female, 52, GP | From the moment I understand that no matter what my response [is], it will not change their way of thinking...I let it go. | À partir du moment où je comprends que, quelle que soit ma réponse, ils ne modifieront pas leur façon de penser, je laisse tomber. |
| P09, Female, 57, Nurse | What made me uncomfortable was also that I didn’t have enough information… [patients] would say “okay, tell me what are the side effects, there are women who aren’t able to have children any more” and I was uncomfortable because I didn’t really know how to respond. | Ce qui m’a rendu mal à l’aise c’est que je n’avais pas assez d’information… [une patiente] m’a dit « Alors dites-moi quels sont les effets secondaires, y a des femmes qui ne sont plus arrivées à avoir d’enfant », j’étais mal à l’aise parce que je ne savais pas trop quoi répondre. |
| P14, Male, 52, Nurse | As a citizen, I do not really agree with vaccinating the youngest [against COVID-19], for example. | En tant que citoyen lambda, j’étais pas forcément en accord avec le fait de vacciner les plus jeunes [contre la COVID-19], par exemple. |
| P04, Male, 41, GP | I know through experience that that doesn’t serve any good to take a strong position that could seem condescending to people who, they themselves are against vaccines. | Je sais d’expérience que ça sert à rien d’avoir une position trop abrupte qui peut paraitre condescendante pour les gens qui, eux, sont contre la vaccination. |
| P15, Male, 35, GP | There are things to put in place and things to say and things to not say…to adapt the discussion…to explain to those who are “convincible”…I think there are people for which there are arguments and things can be done to bring them onto the side of vaccination. | Il y a des choses à mettre en place, des choses à dire et à ne pas dire… pour adapter la discussion… pour expliquer à ceux qui sont « convaincables »… Je pense qu’il y a toute une partie des gens [des patients] pour lesquels des arguments, des choses peuvent être faites pour les ramener du côté de la vaccination. |
| P06, Male, 37, Nurse | It is good because they trust us, and so that helps to speak openly, but sometimes what is bad is that because they treat us like family, sometimes they don’t listen to us. | C’est bien parce qu’ils nous font confiance, et on peut vraiment parler assez librement, par contre ce qui est mauvais c’est que parfois, étant donné qu’ils nous prennent comme la famille, parfois ils nous écoutent plus non plus. |
| P17, Female, 47, Nurse | It was just theory when we talked about [vaccines], when we were in school. That was several years ago and now, [there is] nothing in particular for vaccination. | C’était de la théorie comme quand on a fait les cours [à l’école]. Ça fait quelques années maintenant, je me souviens plus trop, mais en tout cas rien de spécifique sur la vaccination. |
| P03, Female, 28, GP | During [my] internship, I followed a training about motivational interviewing…that can also be used, for example, for tobacco. | Pendant l’internat [stage pendant les études de médecine], j’ai suivi la formation entretien motivationnel… ça peut s’appliquer aussi par exemple sur le tabac. |
| P07, Male, 57, Nurse | We were in contact with pharmacists because they were our intermediaries [with patients]…the pharmacist would say oh well if you have all of these doses do it this way. | On avait un bon contact avec les pharmaciens parce que forcément c’était nos intermédiaires… La pharmacienne me disait regarde si tu as toutes ces doses, fais comme ça. |
| P02, Female, 52, GP | We receive the Revue du Praticien at the practice but I admit I absolutely do not have the time to look into it. | On reçoit la Revue du praticien au cabinet mais je vous avoue que j’ai vraiment pas le temps de mettre le nez dedans. |
| P04, Male, 41, GP | [Motivational interviewing is] something that is new that is now proposed as a training module when we are interns | [L’entretien motivationnel] c’est quelque chose de nouveau qui est maintenant proposé comme module de formation en tant qu’interne. |
| P16, Female, 26, GP | We had some sessions of practical situations with complicated patients. But…it was about other subjects, like about antibiotics for example, or announcing a serious disease. | On a eu quelques séances de mises en scène de situations avec des patients un peu compliqués. Mais à l’époque… c’était d’autres sujets, c’était par rapport aux antibiotiques par exemple, ou l’annonce d’une mauvaise maladie. |
| P05, Female, 27, GP | I think it was good to have a training with role playing and the trainers who explained things well, it was better than learning in books. | Je pense que c’était bien d’avoir une formation avec des jeux de rôle et des formateurs qui nous expliquent bien, c’est peut-être plus simple quand même que l’apprendre dans des livres. |
| P05, Female, 27, GP | I think that [it] helps me sometimes when I don’t have arguments or I feel that the patient is a bit upset, I try to use the basics of the motivational interview to get back on track. And that works pretty well. | Je pense que c’est ça qui m’aide un peu parfois quand je suis à court d’arguments, ou que je sens que le patient est très braqué, j’essaie de me rappeler des bases de l’entretien motivationnel pour un peu pouvoir rebondir. Et ça marche plutôt bien. |
| P18, Male, 30, Pharmacist | I think what would help a lot is learning to identify…[the] nuanced side of patients…Once we know who we are talking to, which personality we are talking to, we can use this or that argument [for vaccination]. | Je pense que ce qui sert beaucoup, c’est ce qui va apprendre à cerner le patient… toutes les personnalités du patient… une fois qu’on sait à qui on parle, à quelle personnalité on parle, on va pouvoir utiliser plutôt tel ou tel argument. |
| P12, Male, 67, GP | [It would be good to have] a roadmap to be able to discuss with people. | [Ça serait bien d’avoir] une feuille de route pour discuter avec les gens. |

*Note.* Quotations are presented here in the order in which they appear in the manuscript.
